# Supplementary figures and images for: Forming individual magnetic biskyrmions by merging two skyrmions in a centrosymmetric nanodisk
Source: Sci Rep. 2019 Jul 2;9:9521. doi: 10.1038/s41598-019-45965-8 (PMC6606756; doi:10.1038/s41598-019-45965-8)

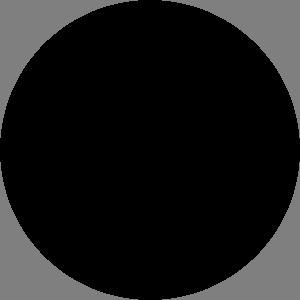

Supplement: Supplementary file 1 — Supplementary Video 1 [file 41598_2019_45965_MOESM1_ESM.gif]

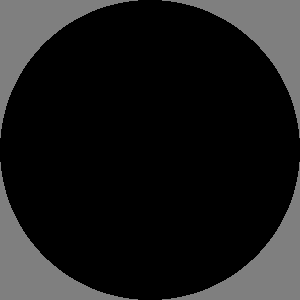

Supplement: Supplementary file 2 — Supplementary Video 2 [file 41598_2019_45965_MOESM2_ESM.gif]

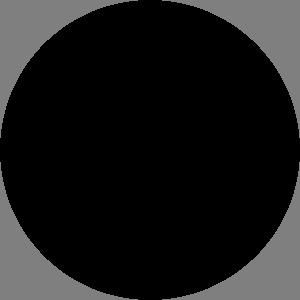

Supplement: Supplementary file 3 — Supplementary Video 3 [file 41598_2019_45965_MOESM3_ESM.gif]
