# Supplementary material for: Forming individual magnetic biskyrmions by merging two skyrmions in a centrosymmetric nanodisk
Source: Sci Rep. 2019 Jul 2;9:9521. doi: 10.1038/s41598-019-45965-8 (PMC6606756; doi:10.1038/s41598-019-45965-8)
Supplement: Supplementary file 4 — Supplementary information [file 41598_2019_45965_MOESM4_ESM.pdf]

# Forming individual magnetic biskyrmions by merging two skyrmions in a centrosymmetric nanodisk
## — Supplementary material —

Börge Göbel,[1] Jürgen Henk,[2] and Ingrid Mertig[1,2]

[1]*Max-Planck-Institut für Mikrostrukturphysik, Halle (Saale), 06120, Germany*
[2]*Martin-Luther-Universität Halle-Wittenberg, Institut für Physik, Halle (Saale), 06099, Germany*

In this Supplementary information we show additional figures which were addressed in the main paper. In Fig. S1 the distance between the two generated skyrmions is changed and the final biskyrmion's orientation is analyzed. In Fig. S2 the helicity of two written skyrmions is chosen equally; a repulsive interaction becomes visible. The figures are further explained in their respective caption.

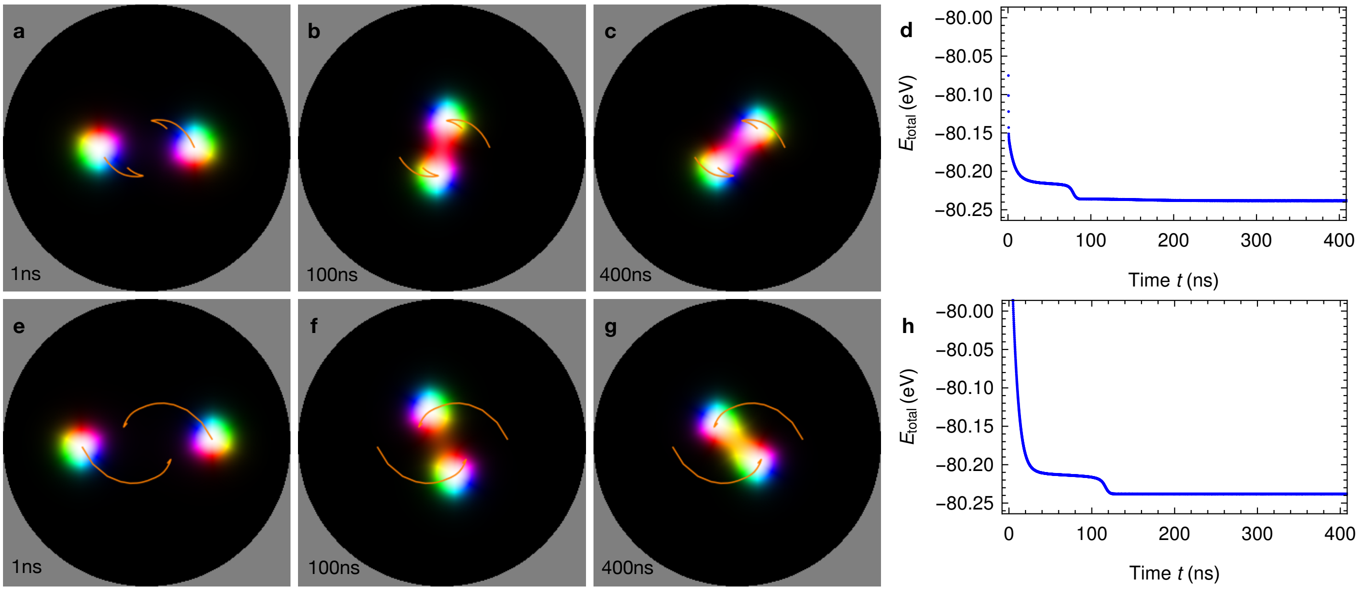


FIG. S1: Formation of a biskyrmion. Like Fig. 5 of the main paper but for a different displacement of the writing devices. In the paper the center's difference was $120\,\mathrm{nm}$. Here, the difference is $100\,\mathrm{nm}$ in the top row and $140\,\mathrm{nm}$ in the bottom row. As is visible in (**a**)–(**c**) the biskyrmion can not overcome the energy barrier originating from the structural lattice (Fig. 2c of the main paper) and relaxes to a different state ($\alpha = 45°$) than in the paper. In the bottom row the final state is the same as in the paper. Even though the starting distance of the two skyrmions is larger, the relaxed biskrmion is in the same energy minimum with an angle of $\alpha = 135°$.

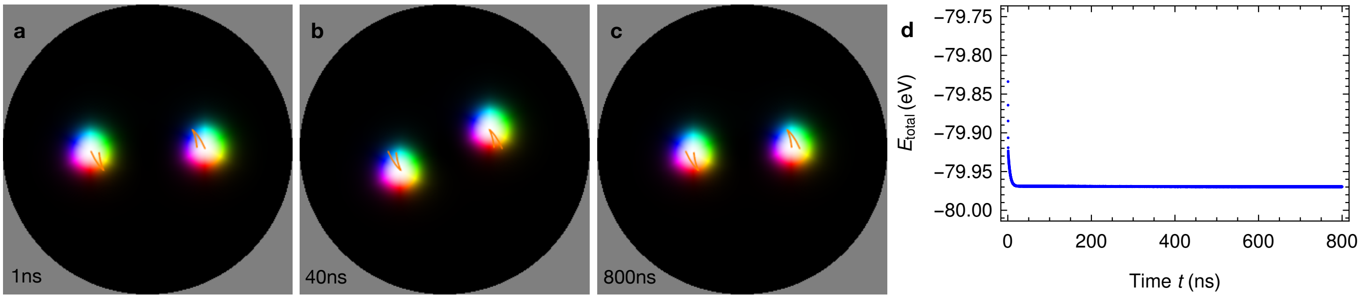


FIG. S2: Repulsion of two identical skyrmions. Like Fig. 5 of the main paper but both skyrmions have the helicity $+\pi/2$. (**a**-**c**) Snapshots and trajectories (orange) of two Bloch skyrmions with the same helicity. The repulsive interaction hinders the spiral motion and the merging process. (**d**) Temporal progress of the system's total energy. During the whole propagation it is higher than the energy of a stable biskyrmion state; cf. Fig. 5d of the main paper.
